# Recent trends in antimicrobial peptide prediction using machine learning techniques

**DOI:** 10.6026/97320630013415

**Published:** 2017-12-31

**Authors:** Yash Shah, Deepak Sehgal, Jayaraman K Valadi

**Affiliations:** 1 Department of Computer Engineering, Thadomal Shahani Engineering College, Mumbai- 400050; 2 Shiv Nadar University, Gautam Budha Nagar, U.P 201314; 3 Center for modelling and simulation, Savitribai Phule Pune university, Pune- 411007

**Keywords:** Antimicrobial peptide, therapeutics, machine learning

## Abstract

The importance to develop effective alternatives to known antibiotics due to increased microbial resistance is gaining momentum in recent years. Therefore, it is of interest to predict, design and computationally model Antimicrobial Peptides (AMPs). AMPs are oligopeptides with varying size (from 5 to over100 residues) having key role in innate immunity. Thus, the potential exploitation of AMPs as novel therapeutic agents is evident. They act by causing cell death either by disrupting the microbial membrane by inhibiting extracellular polymer synthesis or by altering intra cellular polymer functions. AMPs have broad spectrum activity and act as first line of defense against all types of microorganisms including viruses, bacteria, parasites, fungi and as well as cancer (uncontrolled celldivision) progression. Large-scale identification and extraction of AMPs is often non-trivial, expensive and time consuming. Hence, there is a need to develop models to predict AMPs as therapeutics. We document recent trends and advancement in the prediction of AMP.

## Description

Machine learning is considerably applied in different areas of biological knowledge discovery for improved healthcare. Supervised, unsupervised and reinforcement learning are the three major learning methods. Identification of AMPs using a combination of supervised and unsupervised learning techniques is available. A set of experimentally annotated positive AMP peptides collected from the databases is used for supervised learning. The negative data of short peptides collected from a spectrum of available non-secretary peptides is often used for training. A high performance machine learning model is built to classify the data into AMPs and non-AMPs with domain features such as amino acid frequencies and composition extracted from known data using the most suitable performance measures like accuracy, Mathew Correlation Coefficient, ROC etc. Support Vector Machines (SVM), Random Forests (RF) and Artificial Neural networks have been used profusely for the identification of AMPs. SVM employs a linear hyper plane in a higher dimensional feature space for separation. Random forests classifier combines a forest of decision tree models and builds a consensus model. Artificial Neural Networks (ANN) uses interconnected network of neurons.

Thomas et al. (2009) [1], employed supervised learning methods with Support Vector Machines, Random forests and linear discriminant analysis for the identification of AMPs. They judiciously used a combination of composition, physicochemical properties and structural characteristics of amino acids for model building. Conformational similarity, hydrophobicity, normalized van der Waals volume, polarity, polarizability, charge, secondary structure and solvent accessibility of amino acids were used as input features in model building. Subsequently, along with composition, and extracted dipeptide and tripeptide features of the reduced alphabets, the transition and distribution of some of the features along the sequence of peptides were also treated as input attributes to the classifier. Further, they employed SVMRFE (Support Vector Machines Recursive Feature Elimination) and Random Forest Gini Scores for extracting the most informative feature subsets. The model produced a high MCC value of 0.86 for the RF model. A SVM Model with net charge at the physiological pH, ratio between hydrophobic and charged residues, average hydrophobicity (H) and the hydrophobic moment (μH) as features (H and μH were measured based on Eisenberg's hydrophobicity scale) was developed by Porto et al. (2010) [2] on a class of AMP having a cysteine knot motif with three disulfide linkages in their structures. This approach showed 83% accuracy and thus, the first version of CSAMPPRED was made available online. An improved model [3] was later developed with 90% accuracy by eliminating hydrophobic moment as an attribution. An Artificial Neural Networks (ANN) based model was implemented [4] with improved accuracy using physicochemical properties to characterize anti-microbial potency. The new model accommodates peptide aggregation as its salient feature.

AMP's were also predicted elsewhere [5] using multiple alignment followed by subsequent feature selection. A prediction model with high Mathew Correlation Coefficient of 0.73 was developed. Thus, several models are available for the prediction of antiviral, antibacterial or antifungal activities. A CLASSAMP server [6] using SVM and RF models predicting the propensity of a peptide to be antibacterial, antiviral or antifungal is also available. A two level fuzzy K-Nearest Neighbor Model [7] for the prediction of anti-microbial activities of the peptide is available. AMP prediction by a synergistic combination of sequence alignment-SVM- LZ complexity pair wise algorithm [8] is reported. A very high sensitivity value of 95.28% in jackknife test is shown in this model. Anti Hepatitis Peptides [9] identification employing a hybrid combination of Support Vector Machines and Ant Colony Optimization Techniques is interesting. This model provided a 10 fold cross validation accuracy of 94%.

## Conclusion

Databases and prediction servers have a key role in the rational design of novel AMPs as reviewed elsewhere [10]. The workflow starts from collecting information on existing AMPs from available databases for the development of high performance models using most informative domain features. An online server with an ensemble of available models finds application in the development of an AMP with acceptable efficacy. It should be noted that these models are based on sequence features. A hybrid model with a combination of sequence and structure model for the prediction of accurate AMPs is foreseen in the near future.
